# Experimental throughfall reduction barely affects soil carbon dynamics in a warm-temperate oak forest, central China

**DOI:** 10.1038/s41598-017-15157-3

**Published:** 2017-11-08

**Authors:** Haibo Lu, Shirong Liu, Hui Wang, Junwei Luan, Andreas Schindlbacher, Yanchun Liu, Yi Wang

**Affiliations:** 1 Key Laboratory of Forest Ecology and Environment, China’s State Forestry Administration, Institute of Forest Ecology, Environment and Protection, Chinese Academy of Forestry, No. 2 Dongxiaofu, Haidian District, Beijing, 100091 China; 2 International Centre for Bamboo and Rattan, Beijing, 100102 China; 3 Department of Forest Ecology, Federal Research and Training Centre for Forests, Natural Hazards and Landscape-BFW, A-1131 Vienna, Austria; 4 International Joint Research Laboratory for Global Change Ecology, State Key Laboratory of Cotton Biology, College of Life Science, Henan University, Kaifeng, Henan 475004 China

## Abstract

Changing precipitation patterns could affect soil carbon (C) cycling in China’s forests. A throughfall reduction (TFR) experiment was conducted in a warm-temperate oak forest in central China to examine effects of reduced precipitation on total soil respiration (SR), heterotrophic soil respiration (HR), autotrophic soil respiration (AR), soil microbial biomass, and fine root biomass from 2013 to 2016. Rain-out shelters, excluding ~50% of throughfall, were applied between May and September, thereby simulating a ~30% reduction in annual precipitation. Although soil moisture was significantly reduced during TFR, microbial biomass and HR remained unaffected. SR, AR, as well as fine root biomass increased during TFR in a comparable dry year, but remained unaffected during all other years. Annual rates of SR, HR, and AR were all unaffected by TFR. Our results indicate that a mild, steady, reduction in growing season precipitation does not affect soil organic matter decomposition in the oak forest ecosystem studied. Low SR rates during a natural dry-spell indicate that SR can be significantly decreased under more severe drought than imposed by the TFR treatment. Our data suggest a low soil moisture threshold of about 10 vol% for SR in the studied soil.

## Introduction

Northern and north-central China experienced a decrease in mean annual precipitation between 1960–2010^1^ and growing season precipitation is expected to decrease further until 2035 in parts of central China^2^. Covering major parts of central China and ~15% (15.5 × 10^6^ ha) of total forest areas^3^, *Quercus* forests represent the largest vegetation C stock (671 Tg), which accounts for ~18% of all forest vegetation C in China^4^. It yet remains unclear how these important forest ecosystems are affected by decreasing precipitation.

Decreasing precipitation and associated drought can induce tree mortality^5^ thus reduce the C-sink strength of forests^6,7^, or even impose regional forest dieback^8,9^. Moisture limitation can also affect the CO_2_ efflux from soil to the atmosphere (soil respiration; SR)^10–13^ which, after photosynthesis, is the second largest C flux in the global C cycle^14^. Substrate transportation and availability in soil is decreased under water limitation, thus limiting the microbial decomposition of soil organic matter (SOM)^15,16^. As a consequence, heterotrophic soil respiration (HR) can be, at least transiently, reduced^15,17^. Soil water deficit can further impact on plant inner C allocation and plant phenology^18–21^. Such plant driven response will be primarily reflected in the autotrophic soil respiration (AR), which depends on the labile C-flow below ground^22^. The complex interactions between aboveground and belowground processes require distinguishing between drought effects on AR and HR to assess if fast or slow-cycling soil C pools are affected. While drought effects on AR do not directly affect soil C stocks, drought effects on HR can increase or decrease soil C storage^23^.

How reduced precipitation really affects SR can be best studied during natural drought events, but due to the occasional nature of such events, correspondingly long time-series of eco-physiological measurements are required to capture them. Hence, manipulative throughfall-reduction experiments have been suggested as an alternative, straight forward approach to mimic drought and to allow keeping experiments within a reasonable time-frame, while keeping other environmental drivers such as temperature unaffected^24–26^.

With the aim to study the effects of reduced precipitation/throughfall on soil C dynamics of a mesic warm-temperate oak forest, we conducted a large-scale throughfall reduction (TFR) experiment in central China. We sheltered 50% of the forest floor area of three 20 × 20 m plots during the growing-season of 2013–2016 to impose a ~30% reduction of annual throughfall. Effects on SR, AR, HR (estimated by means of trenching), microbial biomass, and fine root biomass were studied. We hypothesized that (I) TFR significantly decreased SR; that (II) HR was primarily affected by TFR because topsoil and litter (both rich in labile C and hosting major parts of the decomposer community) were expected to dry most severely. With this regard, we hypothesized that (III) soil microbial biomass in the topsoil declined during TFR. We further hypothesized that (IV) AR showed less response to TFR because deep-rooting oak trees can extract water from deeper soil layers resulting in generally weaker TFR effects on tree physiology. Fine root biomass was expected to decrease in topsoil as a matter of water shortage^27–29^.

## Results

### Precipitation, soil temperature and soil moisture

**Figure 1:**
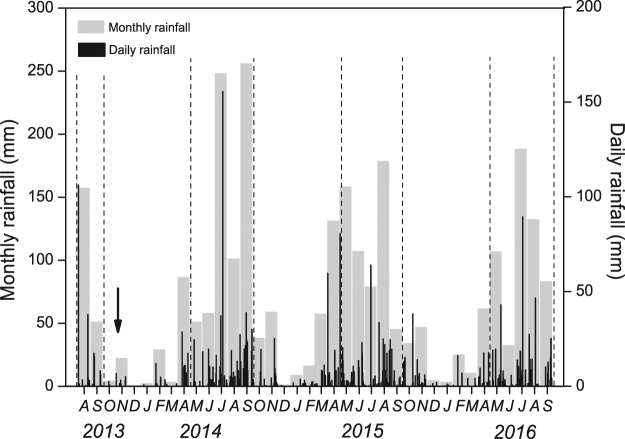
Seasonal variation of daily (black bars) and monthly (grey bars) precipitation under ambient environment. The black arrow shows the natural drought period from September to December in 2013. The areas between two dash lines show the periods when the throughfall was excluded.

**Figure 2:**
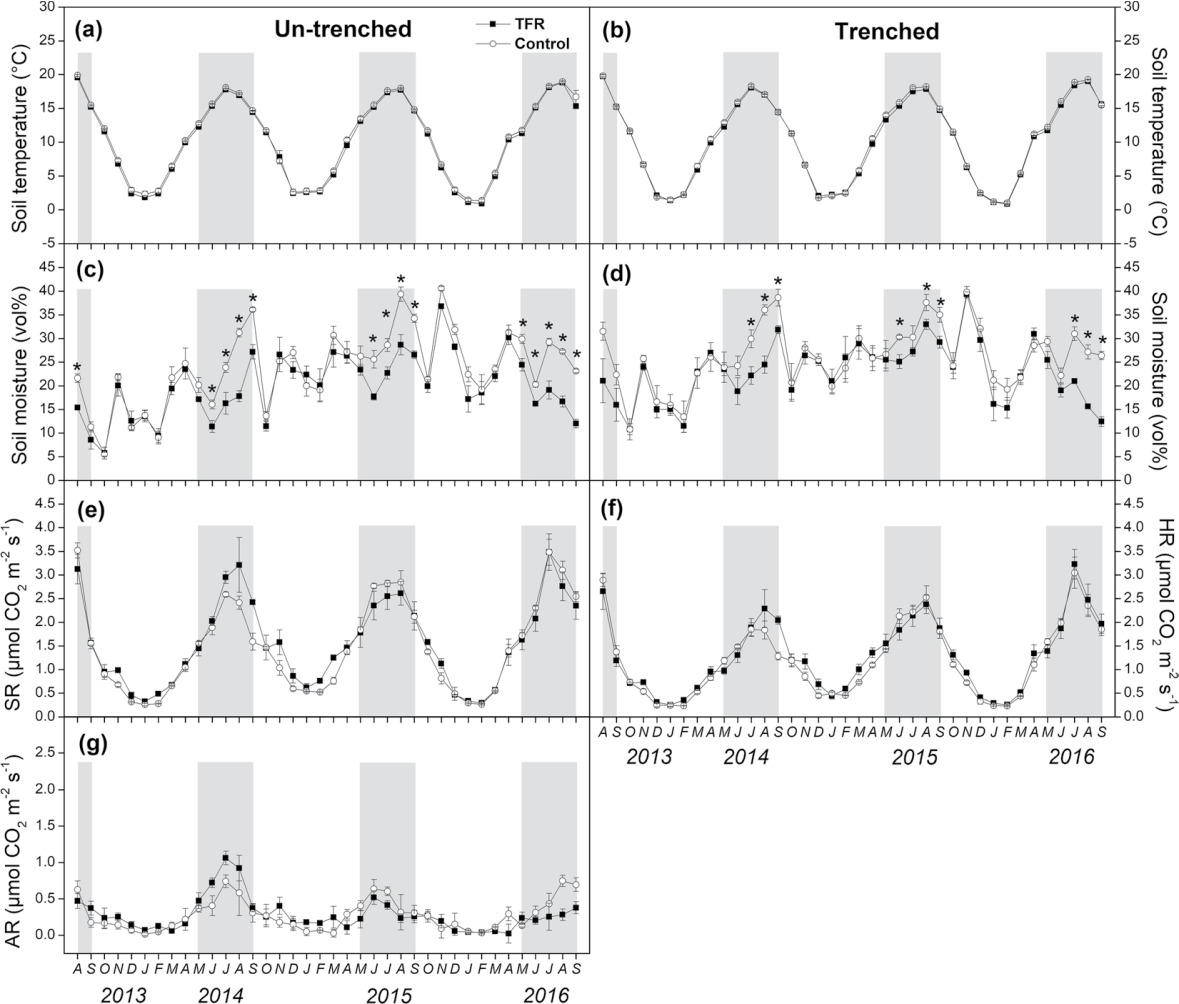
Seasonal course of soil temperature (**a**,**b**), soil moisture (**c**,**d**), total soil respiration (SR; **e**), heterotrophic soil respiration (HR; **f**) and autotrophic soil respiration (AR; **g**) at trenched (**b**,**d**,**f**) and un-trenched (**a**,**c**,**e**,**g**) sub-plots. Symbols show mean values ± SE (n = 3). Asterisks indicate statistically significant differences between control and throughfall reduction (TFR) plots (*P* < 0.05). The shaded areas show the periods when the throughfall was excluded.

Total precipitation showed inter-seasonal variation with >60% precipitation occurring during the growing seasons (May to September) (Fig. 1, Table 1). An extreme rainfall event of 156 mm occurred on July 19, 2014 (Fig. 1), amounting 17% of the annual precipitation in 2014. In 2015 and 2016, rainfall was more evenly distributed throughout the growing seasons (Fig. 1). Low rainfall was recorded between August 2013 and April 2014 resulting in explicitly low soil moisture (<10 vol%) during most of the dormant season 2013/2014 (Figs 1 and 2c). Soil moisture in trenched subplots was less affected by this natural dry period than soil moisture in un-trenched subplots (Fig. 2d).

**Table 1:** Annual precipitation, growing season (May–Sept) precipitation and excluded throughfall at the study site.

Year	Annual precipitation (AP, mm)	Growing season precipitation (GSP, mm)	Excluded throughfall (ΔTF, mm)	ΔTF/AP
2014	927.2	711.8	292.4	31.5%
2015	907.6	565.8	232.4	25.6%
2016	*759 (Jan to Oct)	540.4	222.0	—

ΔTF/AP shows excluded throughfall to annual precipitation. *Indicate the precipitation in 2016 is only from January to October.

Approximately 32% of annual precipitation was excluded by rainout-shelters in 2014 and approximately 26% in 2015 (Table 1). The TFR significantly decreased soil moisture at 0–5 cm soil depth during whole TFR treatment period in un-trenched (*P* = 0.001) and trenched plots (*P* = 0.033; Table 2). Over the entire study period (including dates without TFR), soil moisture was significantly reduced in un-trenched subplots (*P* = 0.007) only (Table 2). TFR decreased soil moisture at 0–5 cm soil depth during shelter-application by on average 4.4 vol% in 2013, 7.5 vol% in 2014, 7.0 vol% in 2015 and 8.3 vol% in 2016 for un-trenched subplots (Fig. 2c, Supplementary Table S1), and by 8.4 vol% in 2013, 6.4 vol% in 2014, 4.0 vol% in 2015 and 8.5 vol% in 2016 for trenched subplots (Fig. 2d, Supplementary Table S1). There was no significant TFR effect on soil moisture in 20 cm, 30 cm and 50 cm soil depth (data not shown). Soil temperature at 0–5 cm depth was unaffected by TFR in both un-trenched and trenched plots throughout the whole study period (Fig. 2a,b).

**Table 2:** Effects of throughfall reduction (TFR), time and their interactions on soil moisture (trenched and un-trenched), soil respiration (SR), heterotrophic soil respiration (HR) and autotrophic soil respiration (AR).

Variable	Source of variation	All TFR periods			Entire study period		
		df	*F*	*P*	df	*F*	*P*
Soil moisture un-trenched	TFR	1	63.17	0.001	1	25.71	0.007
	T	33	52.65	<0.001	75	35.36	<0.001
	T × TFR	33	4.46	<0.001	75	3.49	<0.001
Soil moisture trenched	TFR	1	10.28	0.033	1	3.28	0.144
	T	33	20.54	<0.001	75	27.94	<0.001
	T × TFR	33	3.00	<0.001	75	3.37	<0.001
SR	TFR	1	<0.001	0.99	1	0.36	0.581
	T	33	21.57	<0.001	75	50.54	<0.001
	T × TFR	33	2.16	0.001	75	1.57	0.008
HR	TFR	1	0.06	0.815	1	0.53	0.508
	T	33	27.01	<0.001	75	56.50	<0.001
	T × TFR	33	1.75	0.015	75	1.27	0.101
AR	TFR	1	0.52	0.512	1	0.10	0.763
	T	33	3.83	<0.001	75	5.59	<0.001
	T × TFR	33	2.40	<0.001	75	2.09	<0.001

Repeated measures ANOVA, n = 3.

### Soil CO_2_ efflux

**Figure 3:**
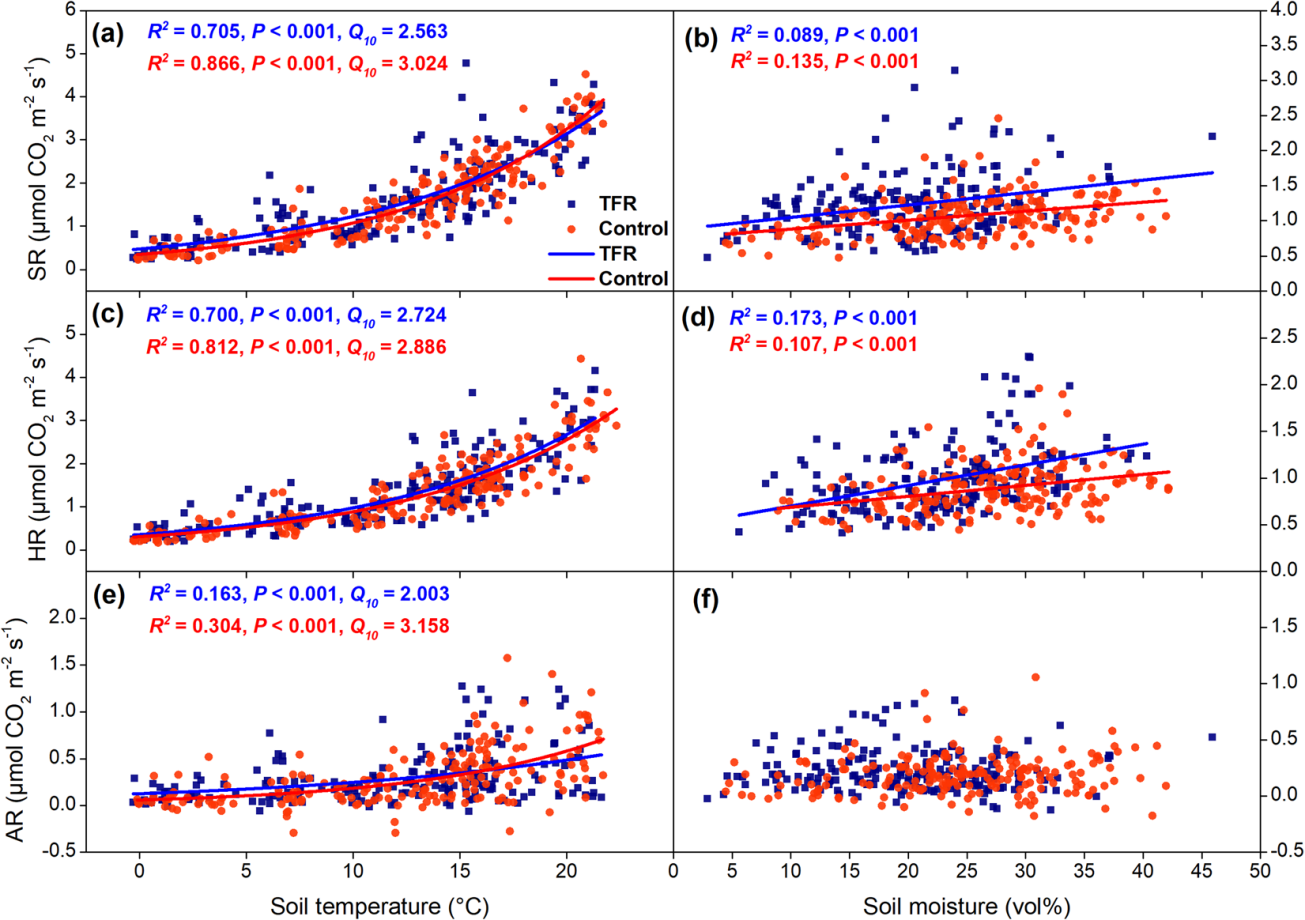
Dependency of soil CO_2_ efflux (SR; **a**), heterotrophic soil respiration (HR; **c**) and autotrophic soil respiration (AR; **e**) to soil temperature at 0–5 cm depth (equation (1)); Dependency of temperature (10 °C) normalized SR (**b**), HR (**d**) and AR (**f**) to soil moisture. Blue dots and lines indicate throughfall reduction treatment (TFR), and red dots and lines indicate control treatment.

The SR, HR and AR closely followed the seasonal course of soil temperature at 0–5 cm depth in both control and TFR plots (Fig. 2). Soil temperature explained 71%, 70% and 16% variation of SR, HR and AR under TFR treatment, respectively; and explained 87%, 81% and 30% variation of SR, HR and AR under control treatment, respectively (Fig. 3a,c,e). Temperature normalized SR and HR significantly, and positively, correlated with soil moisture at 0–5 cm depth, while no significant correlation was observed between temperature-normalized AR and soil moisture (Fig. 3b,d,f).

The SR at control and TFR plots was ~ 30% lower (control: 0.86 ± 0.26 μmol CO_2_ m^−2^ s^−1^; TFR: 0.99 ± 0.22 μmol CO_2_ m^−2^ s^−1^) during the dry period in autumn 2013, when compared with the same months in 2014 (control: 1.17 ± 0.23 μmol CO_2_ m^−2^ s^−1^; TFR: 1.57 ± 0.32 μmol CO_2_ m^−2^ s^−1^) and 2015 (control: 1.20 ± 0.36 μmol CO_2_ m^−2^ s^−1^m^−2^ s^−1^; TFR: 1.33 ± 0.35 μmol CO_2_ m^−2^ s^−1^).

**Figure 4:**
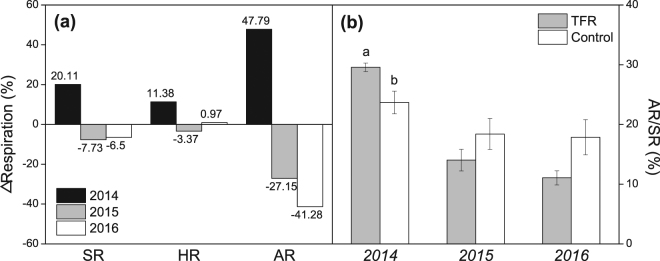
(**a**) Throughfall reduction (TFR) effects on relative changes (%) of soil CO_2_ efflux (SR), heterotrophic soil respiration (HR) and autotrophic soil respiration (AR) during shelter application from May to September. (**b**) Relative contribution (%) of AR to SR at control and TFR plots (TFR from May to September). Symbols show mean values ± SE (n = 3). Different letters indicate significant difference between treatments (*P* < 0.05).

TFR differently affected SR during individual study years. During TFR, SR increased by on average 20% in 2014, though the increase was statistically insignificant (see Supplementary Table S1). SR insignificantly declined during TFR in 2015 and was not affected at all in 2016 (Figs 2 and 4a, Supplementary Table S1). TFR did not affect HR during the whole study (Table 2). TFR increased AR by 48% during rainout-shelter application in 2014, and decreased AR by 27% and 41% during rainout-shelter application in 2015 and 2016 (Figs 2 and 4a), but the effects of TFR on AR were statistically insignificant as well (Table 2). The relative contribution of AR to SR (AR/SR) under TFR was significantly (5.9%) higher than that in control plots in 2014 (*P* = 0.044; Fig. 4b). We did not observe any distinctive, rewetting effects on SR, HR, or AR after rainout-shelter removal. Overall, TFR did not affect annual cumulative SR, HR, and AR which were nearly identical between control and TFR (Table 3). Among the two years with a full seasons record, cumulative annual SR and HR under control treatment in 2014 were significantly lower than that in 2015 (*P* = 0.03 for SR, *P* = 0.02 for HR) while cumulative AR of TFR plots was significantly higher in 2014 than in 2015 (*P* = 0.03; Table 3).

**Table 3:** Cumulative annual soil CO_2_ efflux (SR), heterotrophic soil respiration (HR) and autotrophic soil respiration (AR) under throughfall reduction (TFR) and control treatments during field seasons 2014 and 2015.

Field season	Total cumulative flux under TFR (ton C ha^−1^)			Total cumulative flux under control (ton C ha^−1^)		
	SR	HR	AR	SR	HR	AR
**2014**	5.67 ± 0.43	4.17 ± 0.39	1.50 ± 0.12 **a**	4.97 ± 0.22 **a**	3.88 ± 0.16 **a**	1.09 ± 0.38
**2015**	5.76 ± 0.64	4.89 ± 0.50	0.86 ± 0.15 **b**	6.03 ± 0.21 **b**	5.06 ± 0.29 **b**	0.97 ± 0.27

Different lowercase letters indicate significant difference between field seasons (*P* < 0.05). There was no significant treatment (TFR) effect. Data show mean values ± SE (n = 3).

### Fine roots biomass and microbial biomass

**Figure 5:**
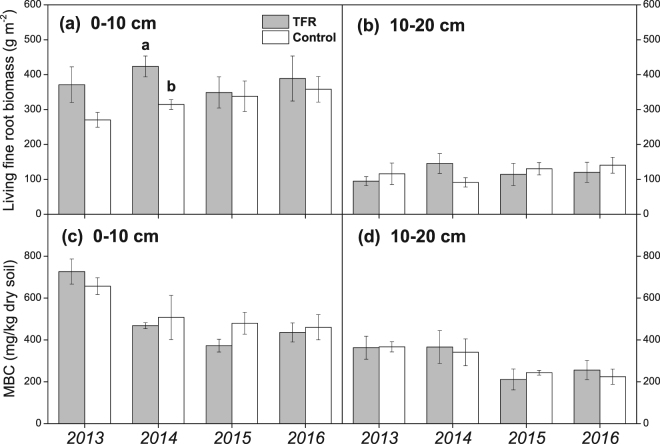
Living fine root biomass (**a**,**b**) and microbial biomass (**c**,**d**) under throughfall reduction (TFR) and control treatments in 0–10 and 10–20 cm soil depth. Grey columns indicate TFR treatment, and white columns indicate control treatment. Different letters indicate significant difference (*P* < 0.05). Symbols show mean values ± SE (n = 3).

Living fine root biomass in 0–10 cm soil depth was significantly higher at TFR plots than that of control in 2014 (*P* = 0.03; Fig. 5a), but no TFR effects on fine root biomass were observed in 2015 and 2016. In the depth of 10–20 cm soil, no significant differences were found in fine root biomass between TFR and control treatments (Fig. 5b). In both 0–10 and 10–20 cm soil depth, there were no significant difference in microbial biomass C between control and TFR plots, but microbial biomass C in 0–10 cm depth soil was higher in 2013 than during the following years (Fig. 5c,d).

## Discussion

Although TFR had significantly reduced soil moisture in the topsoil, soil CO_2_ efflux, microbial biomass and fine root stocks were barely affected. In contradiction to our hypotheses, HR and microbial biomass remained largely unaffected by TFR. The SR and AR showed short-term temporal responses during rainout-shelter application, which, however, were not reflected in their annual budgets.

Contradicting to our hypotheses, SR increased during TFR in 2014. The increase in SR resulted from a strong increase in AR at TFR plots, which was accompanied by significantly higher fine root biomass in the topsoil. Very similar observations (no HR response, but increasing RA) were observed by Metcalfe *et al*.^30^ who conducted a TFR experiment in a tropical forest in the Amazon. At our site, the higher fine root biomass in 2014 likely was due to the naturally preceding drought conditions during autumn and winter 2013 and the comparatively low soil moisture before and during rain-out shelter application in 2014. Soil water deficit can enhance plant C allocation belowground and stimulate fine root production to increase water uptake^20,31^. Such stimulated fine root growth by soil water shortage was observed in mature Douglas-fir forest in western Oregon^32^ and deciduous mixed forest dominated by *Fagus* and *Quercus* in north-west Germany^33^. Increased fine root biomass in soil has been shown to increase the contribution of AR^34,35^. This is a likely explanation for the unexpected increase in SR during TFR in 2014. The triggering effect of TFR on SR and AR disappeared during the following year 2015, during which soil moisture was higher than the year before at control as well as TFR plots and fine root biomass became similar again. The different response of fine root biomass and AR to TFR between wet and dry years highlights a general limitation of TFR approaches. Roofs impose soil drought, while atmospheric conditions (e.g. vapor pressure deficit) in the tree crowns remain largely unaffected. Since C allocation in trees is closely linked to soil C cycling^18,36,37^, the inability of TFR to fully mimic drought conditions can impose methodological bias^38^. The first study year, which was characterized by naturally dry conditions, may therefore have best predicted the full ecosystem response to TFR.

The TFR had no significant effect on HR and microbial biomass throughout our study, suggesting that decomposer communities were well adapted to dry conditions at the site. This is in line with observations from a previous study at the same site^39^ using the same trenching technique, but applying much smaller (4 × 5 m) roofs. As in our study, HR was unaffected by TFR, though soil moisture of trenched plot in the study of Liu *et al*.^39^ never reached such low values as in our TFR treatment. Our observations were also consistent with Hinko-Najera *et al*.^13^ who reported that 40% TFR had no distinct effects on HR in a dry temperate *eucalypt* forest in Australia. In contrast, Borken *et al*.^23^ found that throughfall exclusion primarily reduced HR in a temperate forest dominated by red maple and red oak. Muhr and Borken^40^ reported that throughfall exclusion significantly decreased HR but had negligible effects on AR in a temperate Norway spruce forest in south Germany. Another potential reason for the overall weak response of HR could be the fact that the mild TFR treatment had dried out the topsoil, while deeper soil layers remained unaffected. Increasing contribution of HR from deeper soil layers was observed in a TFR experiment in the tropics^11^. However, C contents in deeper soil layers were much higher in the tropical soil^11^ than at our site^41^ and soils at our site were comparatively shallow. Along with soil C, microbial biomass declined with depth (Fig. 5 and You *et al*.^41^), suggesting that the contribution of HR from deeper layers was minor at our site. The ability of soil microbes to maintain HR at low soil moisture contents, as it was observed in our study, could also be related to soil texture. Moisture thresholds have been shown to generally becoming lower with decreasing clay contents in a comparison of TFR experiments^42^, suggesting a low moisture threshold at the sandy soil studied. Since moisture conditions have never substantially limited HR in our trenched plots, an actual value for the lower moisture threshold of HR could not been derived with the trenching approach. SR rates during the natural dry period in autumn 2013 can, however, give some hints. SR was significantly suppressed during this natural dry period. If HR and AR were equally suppressed^43^, the soil moisture threshold at our site would have been around 10 vol% for HR. This is in the same range as reported by Luan *et al*.^44^, who observed suppressed rates of HR during a dry period (~10 vol% soil moisture) in a soil translocation experiment which was conducted close to our site. Similarly low threshold values (~12 vol% soil moisture) for SR were observed at Harvard temperate hardwood forest^45^ and in a boreal aspen forest^46^, respectively. Higher (~20 vol%) soil moisture thresholds for HR were reported for a Bhutan oak forest^47^ and an oak forest in central Italy^48^.

With regard to the applied trenching method, it further has to be noted that the estimated growing season contribution of AR to SR was low (10–30%), when compared to other studies in similar ecosystems. Bond-Lamberty *et al*.^49^ synthesized published soil respiration data from 54 forests and indicated that the relative contribution of AR to SR ranged from 28% to 62% in warm-temperate forests. The low AR estimate at our site was likely a matter of the caveats of the trenching method. Trenching typically increases the soil moisture content^50–52^ as it was also the case in our study. The difference in soil moisture between un-trenched and trenched plot soil moisture was, however, comparably low (~1–6 vol%). Because TFR decreased soil moisture at trenched plots only to a minimum of ~10 vol% (when compared to ~5 vol% at un-trenched plots), we cannot fully guarantee that our AR estimates were without any bias during water contents below 10 vol%. During moisture contents >10 vol%, the method should have provided more robust results. Decomposition of dead, cut-off fine roots can add to HR at trenched plots, especially during the first year after trenching^50^. The additional CO_2_ efflux from decomposing fine roots typically results in underestimated AR contributions. As we did not consider such fine root decomposition effects, we provide a rather rough, and potentially low quantitative estimate of AR in our study. Nevertheless, the effects of TFR on general patterns of SR, HR, and AR should have been adequately resembled in our experimental setup since the quantitative trenching-bias was the same at control and TFR treatments.

He *et al*.^53^ estimated that the forest vegetation C stock of China will increase by 14 Pg C from 2010 to 2050, and that deciduous broadleaf forest could contribute ~40%. Thus, as a main contributor, *Quercus* forests will play a vital role in regulating regional C sequestration and forest C budgets in central China. Our observations indicate that mildly decreasing growing season precipitation likely has little effect on soil C dynamics in mesic oak forest ecosystems at the climatic transitional zone. The observed decrease of the soil CO_2_ efflux during a natural dry spell shows that more severe future drought can decline SOC decomposition, at least on the shorter-term.

## Materials and Methods

### Study site

The experimental sites are located at the Forest Ecological Research Station in the Baotianman Natural Reserve (30°20′–33°36′N, 111°47′–112°04′E), Henan province, central China, at a mean altitude of 1400 m a.s.l.. The climate is northern transitional subtropical to warm-temperate, with a mean annual air temperature of 15.1 °C and a mean annual precipitation of 890 mm^39^. Over 60% of rain falls in the growing season (May–Sept)^54^. The soils are dominated by Haplic Luvisol^55^, with 27–30% clay, 11–13% slit and 57–62% sand content and an average depth of 40–60 cm. The soil pH in our plots ranged from 4.4–5.1 and the soil organic carbon stock at 0–10 cm mineral soil depth was estimated as ~40 ton C ha^−1^. The experiment was conducted in a 60 years old secondary forest dominated by *Quercus aliena* var. *acuteserrata*. Mean stem diameter at breast height (DBH), and stem density were 19.2 cm and 1913 trees ha^−1^, respectively. Over 80% of the fine root biomass was concentrated between 0–30 cm mineral soil depth^55^.

### Throughfall reduction (TFR) setup

Six 20 m × 20 m plots with similar stand and site conditions were established and three were selected randomly for TFR (see Supplementary Fig. S1). To avoid pseudo-replication^56^, each replicate separated from one another at least 300 m. Prior imposing the TFR treatment, soil physical and chemical properties were assessed to identify the original divergences. This work was carried out following the Forestry Standards “Observation Methodology for Long-term Forest Ecosystem Research” of China (LY/T 1952–2011).

TFR was accomplished by intercepting throughfall with approximately 160 shelter-panels (0.5 m × 3 m) suspended in 1.5 m–2.5 m above the forest floor (see Supplementary Fig. S2). Each panel consisted of two parallel 3 m steel tubes covered by transparent plastic sheet with a width of 0.5 m. All the panels were installed perpendicular to the slope direction, and kept at 2.5 m high for one sides and 1.5 m for the other in order to drain water into gutters. Two rectangular stainless gutters (0.4 m × 20 m) were placed at 1.5 m high throughout each treatment plot to collect and conduct the water out of the plot. The shelter treatments resulted in a ~ 50% reduction in throughfall reaching the forest floor during application. Throughfall was excluded during the growing seasons (May–Sept.) from 2013 until 2016. Litter fallen on the panels was collected and evenly spread back on the plots forest floor fortnightly. To prevent the potential lateral water movement and surface runoff from surrounding forest, a 0.7 m deep trench around the plot boundary was excavated and lined with 5 mm thick plastic plates in March 2013. All measurements in the plots were carried out at least 3 m buffer distance from the trench edge to exclude edge effects.

### Soil CO_2_ efflux and microclimate

The trenching method was applied to partition HR from SR. Ten 3 m × 3 m subplots were randomly set at each plot. Five subplots were trenched (HR) and the other 5 served as un-trenched control (SR).Trenches around the subplot boundary were excavated to the depth of ~1 m and lined with 5 mm thick plastic plates to prevent root in-growth in March 2013. All plants in the trenched subplots were removed in weekly intervals. To avoid initial disturbance effects from shelter installation and trenching, we started CO_2_ efflux measurements four months after plot setup (in August 2013).

In the central zone of each un-trenched and trenched subplot, a 8 cm high PVC collar (19.6 cm inner diameter) was placed (slightly inserted into the mineral soil) to serve as permanent base for soil CO_2_ efflux measurements. A Li-8100 soil CO_2_ flux system (LI-COR Inc., Lincoln, NE, USA) was employed to measure the soil surface CO_2_ efflux. A three minutes measurement cycle was carried out for each collar. During each measurement cycle, the measurement chamber was closed for 105 s (including 15 s dead band time and 90 s data logged time). CO_2_ concentrations were collected at the rate of 1 Hz last for 90 s. The CO_2_ efflux rate was calculated by fitting an exponential function to the chamber headspace CO_2_ increase. The measurements were taken twice per month during the whole experimental period.

Total precipitation data was automatically collected using a RR-9100 multi parameter automatic weather station (Rainroot Inc., Beijing, China) at Forest Protection Station which was about 300–500 meters away from the individual plots. The canopy interception percentage of oak forests in our study site was estimated to be ~18%^57^. Based on this, we acquired the total amount of throughfall during the growing seasons. Soil temperature and moisture of three points around each collar was determined simultaneously with the soil CO_2_ efflux measurement. Soil temperature (0–5 cm mineral soil depth) and moisture (0–5 cm mineral soil depth) were manually measured by a portable temperature probe connected with the Li-8100 and a portable time domain reflectometer MPKit-B soil moisture gauge (NTZT Inc., Nantong, China), respectively. In addition, in each plot, an Em50 data loggers equipped with four 5TM combined soil temperature and moisture probes (Decagon Devices Inc., Pullman, WA, USA) was employed to continuously measure soil temperature and moisture at a depth of 0–5 cm for both un-trenched and trenched subplots at 30 min interval.

### Soil microbial biomass and fine root biomass

We sampled soil cores for microbial biomass and fine root biomass determination in August 2013, 2014, 2015 and 2016. In each plot, five soil cores were taken along the diagonal direction at 0–10 cm and 10–20 cm depth, using a soil auger with 6.8 cm inner diameter. Subsequently, the five soil cores were merged into a mixed sample. Fine roots (<2 mm) were manually collected from each mixed soil sample and fresh soil was stored in ice chest during transport to the laboratory^35^. Fresh soil samples were sieved by 2 mm mesh for further analysis.

The chloroform fumigation–extraction method was employed to determine the soil microbial biomass C (MBC)^58^. Ten grams of fresh soil were fumigated with CHCl_3_ for 24 hours at 25 °C, meanwhile 10 g of un-fumigated soil from the same sample were kept at 25 °C for 24 h. Fumigated and un–fumigated samples were extracted with 40 ml of 0.5 M K_2_SO_4_ and shaken for 40 minutes. The extracted solution was filtered for total organic carbon (TOC) determination using a Vario TOC analyzer (Elementar Analysensysteme, Langenselbold, Germany). MBC was calculated from the differences of K_2_SO_4_ - extracted TOC contents between fumigated and un–fumigated soil applying a conversion factor *k*_*EC*_ of 0.45^58^.

Collected fine roots (<2 mm diameter) were sieved by 2 mm mesh and washed with distilled water for further analysis. Washed fine roots were manually grouped into living roots and necrotic roots according to the color and morphology^59^. Then, all root samples were dry at 65 °C in an oven for 72 hours to constant weight for root biomass determination^60^.

### Data analysis

Autotrophic soil respiration (AR) was calculated as the difference between total soil respiration (SR) and heterotrophic soil respiration (HR). Cumulative annual SR and HR (ton C ha^−1^) of each plot was calculated by linear interpolation between measurement dates of the corresponding year (OriginPro 8.5, procedure Interpolation and Integrate). Cumulative AR was calculated as the differences between cumulative SR and cumulative HR.

We fitted an exponential function between field soil temperature and SR, HR and AR^61^:

1 $$R={R}_{10}\times {{Q}_{10}}^{((T-10)/10)}$$

where *R* and *T* are the measured soil CO_2_ efflux rate (μmol CO_2_ m^−2^ s^−1^) and corresponding measured soil temperature (°C) at 0–5 cm soil depth; *Q*_10_ is the apparent temperature sensitivity and *R*_10_ is the basal soil respiration rate (μmol CO_2_ m^−2^ s^−1^) at 10 °C.

We normalized all CO_2_ efflux measurements to a soil temperature of 10 °C for further analyzes of soil moisture effects on SR, HR and AR:

2 $${R}_{10Norm}=R\times {{Q}_{10}}^{((10-T)/10)}$$

where *R* and *T* are the measured respiration rate of SR and HR (μmol CO_2_ m^−2^ s^−1^) and corresponding measured soil temperature (°C) at 0–5 cm soil depth, respectively; *Q*_10_ were acquired through equation (1). *R*_10Norm_ is the soil CO_2_ efflux rate normalized to 10 °C soil temperature (μmol CO_2_ m^−2^ s^−1^). Normalized AR was calculated as the difference between normalized SR and normalized HR.

Linear regression was employed to examine the effects of soil moisture on normalized SR, HR and AR. Independent-samples *t*-test was employed to examine the impacts of TFR on fine root biomass, microbial biomass carbon, the relative contribution of AR to SR (AR/SR). Annual cumulative soil CO_2_ efflux was compared using independent-samples *t*-test as well. Repeated measures ANOVA was used to investigate the effects of TFR on soil moisture, SR, HR and AR over the all TFR dates, as well as over the entire study period. Where soil moisture, SR, HR and AR (measure variables) were repeated measured over time, the independent variable was the measurement date. When significant differences were found (*P* < 0.05), independent-samples *t*-tests were used to test which months differed significantly (asterisks in Fig. 2). All data were examined for assumptions of normality and homogeneity before these analyses were performed. Statistical analyses were carried out using IBM SPSS Statistics Version 20.0 (IBM Corporation, New York, USA).

### Data availability statement

All data associated with the current study are available from the corresponding author on reasonable request.

## Electronic supplementary material

Supplementary Information
