# Supplementary material for: Experimental throughfall reduction barely affects soil carbon dynamics in a warm-temperate oak forest, central China
Source: Sci Rep. 2017 Nov 8;7:15099. doi: 10.1038/s41598-017-15157-3 (PMC5678107; doi:10.1038/s41598-017-15157-3)
Supplement: Supplementary file 1 — Supplementary Information [file 41598_2017_15157_MOESM1_ESM.pdf]

Supplementary Information

Experimental throughfall reduction barely affects soil carbon dynamics in a warm-temperate oak forest, central China

Haibo Lu[1], Shirong Liu*[,1], Hui Wang[1], Junwei Luan[2], Andreas Schindlbacher[3], Yanchun Liu[4], Yi Wang[2]

[1] *Key Laboratory of Forest Ecology and Environment, China's State Forestry Administration, Institute of Forest Ecology, Environment and Protection, Chinese Academy of Forestry, No.2 Dongxiaofu, Haidian District, Beijing, 100091, China*

[2] *International Centre for Bamboo and Rattan, Beijing 100102, China*

[3] *Department of Forest Ecology, Federal Research and Training Centre for Forests, Natural Hazards and Landscape-BFW, A-1131 Vienna, Austria*

[4] *International Joint Research Laboratory for Global Change Ecology, State Key Laboratory of Cotton Biology, College of Life Science, Henan University, Kaifeng, Henan 475004, China*

First author: **Haibo Lu** Email: luhb87@163.com

Corresponding author: **Shirong Liu**

Tel: (86)10-62889311, Fax: (86)10-62884229

Email: liusr@caf.ac.cn

Figure S1

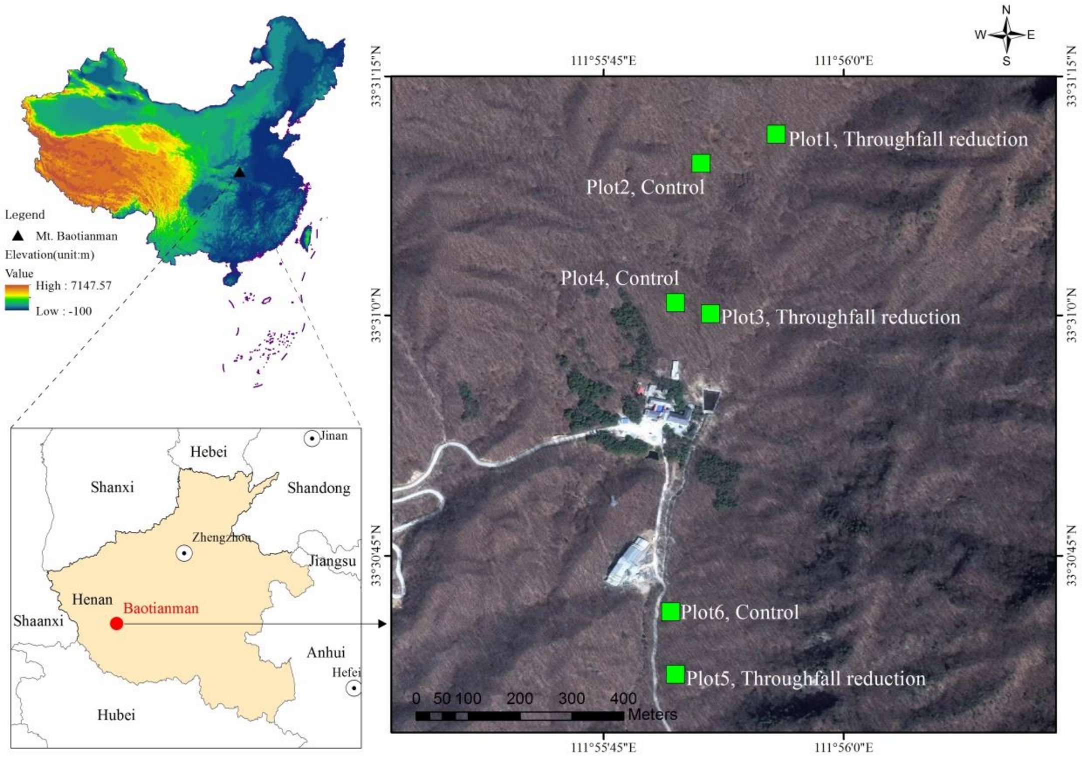

Figure S1. Map of the study area and the layout of the six experimental plots (20 m × 20 m). The map was created using ESRI ArcGIS, ArcMap 10.2 (http://www.esrichina.com.cn/softwareproduct/ArcGIS/). The satellite photograph was free downloaded through ArcGIS 10.2 software. Source: Esri, DigitalGlobe, GeoEye, Earthstar Geographics, CNES/Airbus DS, USDA, USGS, AEX, Getmapping, Aerogrid, IGN, IGP, swisstopo, and the GIS User Community. For more information on this satellite photograph, including the terms of use, please visit: http://goto.arcgisonline.com/maps/World_Imagery.

Figure S2

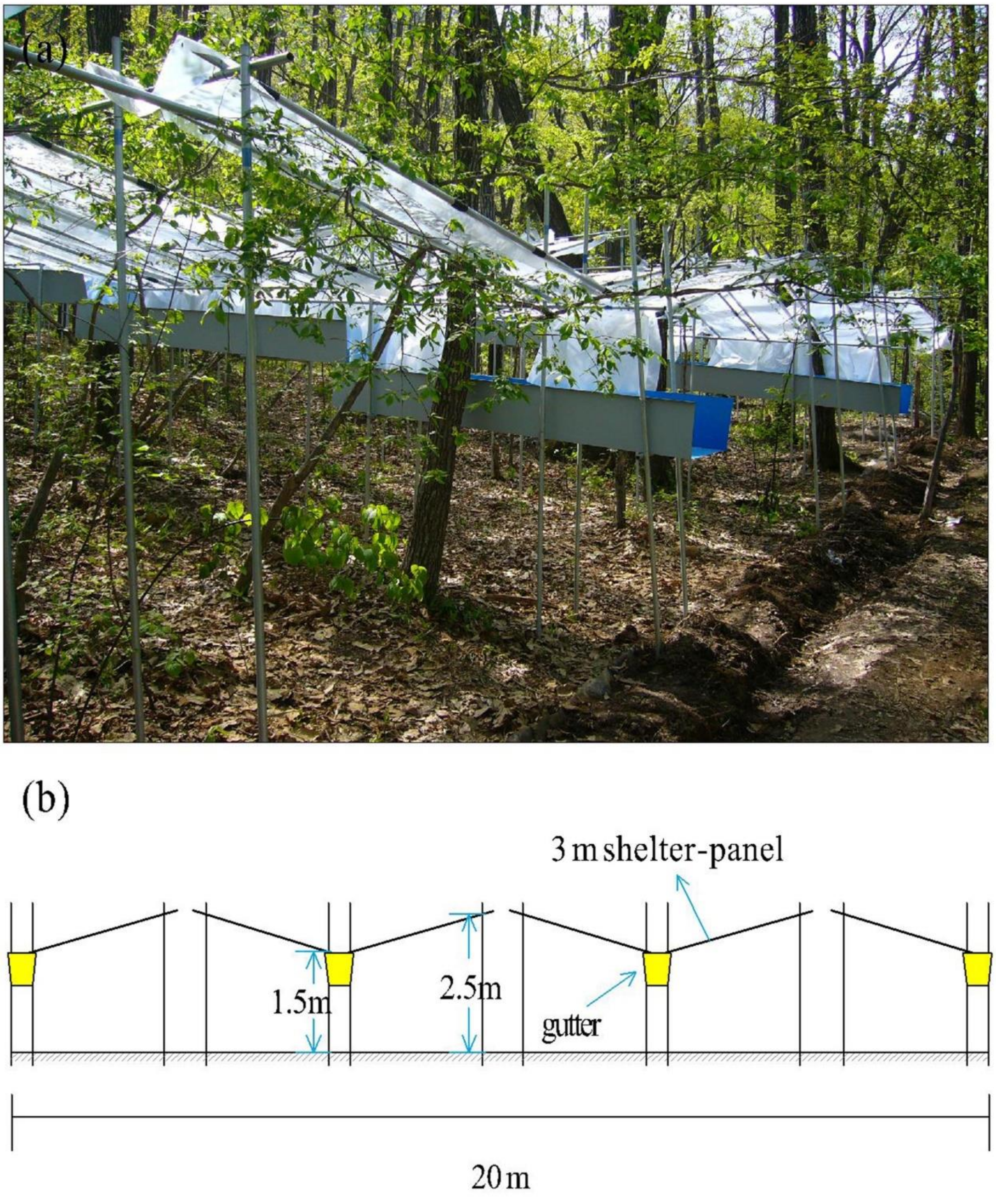

Figure S2. (a) Image of rain-out shelters in one of three plots.

(b) Horizontal view of the rain-out shelter constructions.

Table S1 Effects of throughfall reduction (TFR) treatment during individual growing season, time and their interactions on soil moisture (trenched and un-trenched), soil respiration (SR), heterotrophic soil respiration (HR) and autotrophic soil respiration (AR), n = 3.

Variable	Source of variation	2014 (May – Sept)			2015 (May - Sept)			2016 (May - Sept)		
		df	*F*	*P*	df	*F*	*P*	df	*F*	*P*
**Soil moisture un-trenched**	**TFR**	1	38.1	0.004	1	35.1	0.004	1	251	< 0.001
	**T**	9	59.5	< 0.001	9	21.2	< 0.001	9	44.1	< 0.001
	**T×TFR**	9	6.98	< 0.001	9	1.92	0.091	9	5.31	< 0.001
**Soil moisture trenched**	**TFR**	1	5.98	0.071	1	3.40	0.139	1	52.1	0.002
	**T**	9	56.3	< 0.001	9	13.2	< 0.001	9	41.2	< 0.001
	**T×TFR**	9	7.9	< 0.001	9	0.90	0.531	9	7.99	< 0.001
**SR**	**TFR**	1	3.54	0.133	1	0.31	0.609	1	0.39	0.568
	**T**	9	8.82	< 0.001	9	26.9	< 0.001	9	46.2	< 0.001
	**T×TFR**	9	1.70	0.125	9	1.08	0.401	9	0.85	0.578
**HR**	**TFR**	1	1.01	0.372	1	0.03	0.864	1	0.05	0.841
	**T**	9	6.69	< 0.001	9	38.9	< 0.001	9	48.9	< 0.001
	**T×TFR**	9	2.02	0.065	9	1.19	0.336	9	1.50	0.186
**AR**	**TFR**	1	2.70	0.176	1	2.84	0.167	1	11.8	0.026
	**T**	9	4.21	0.001	9	1.89	0.097	9	1.32	0.260
	**T×TFR**	9	0.79	0.632	9	1.62	0.16	9	3.43	0.004
